# Evaluation of a creatinine-based equation to predict urine volume in nonpregnant, nonlactating Holstein cows

**DOI:** 10.3168/jdsc.2025-0846

**Published:** 2026-01-16

**Authors:** J.L. Bermeo, L.C. Solórzano, A. Rico, N. Silva-del-Río

**Affiliations:** 1 Veterinary Medicine Teaching and Research Center, University of California–Davis, Tulare, CA 93274; 2 Department of Animal Science, University of Puerto Rico–Mayagüez, Mayagüez, PR 00680; 3 GLC Minerals LLC, Green Bay, WI 54303; 4 Department of Population Health and Reproduction, School of Veterinary Medicine, University of California–Davis, Davis, CA 95616

## Abstract

Accurate estimation of urine output is essential for assessing nutrient utilization, particularly for nutrients predominantly excreted through urine, such as minerals and protein-derived metabolites. An equation utilizing creatinine concentration and BW has been previously developed to estimate urinary volume (EUV) in lactating Holstein dairy cows, defined as EUV = [29 × BW (kg)]/[urinary creatinine (mg/L)]. Our objective was to evaluate whether this equation introduces bias when used for estimating urinary output in nonpregnant, nonlactating Holstein cows and to identify factors influencing its accuracy. For our study, we used 72 paired observations that included observed urinary volume (OUV) and urinary creatinine concentration. These observations were obtained from a prior research study that assessed the relative availability of various magnesium (Mg) sources using a duplicated 6 × 6 Latin square design, with cows (n = 12) grouped into squares based on lower (square 1) or higher (square 2) BW across 6 periods. Enrolled cows were of second parity (n = 8) and third or greater parity (n = 4), with BW ranging from 590 to 831 kg. To determine the agreement between EUV and OUV, we constructed 2 mixed-effects models. The first model evaluated slope bias (testing if the slope was significantly different from 1), whereas the second assessed mean bias (testing if the intercept was significantly different from 0) between EUV and mean-centered OUV. In our dataset, creatinine excretion per kilogram of BW ranged from 15.0 to 35.6 mg/kg BW with an average of 27.6 mg/kg BW. When assessing the agreement between OUV and EUV, we observed both slope and mean biases when applying the creatinine-based equation. Furthermore, there was a bias estimate across BW quartiles. Overall, 76.4% of observations fell within ±10% deviation range between EUV and OUV. These findings suggest that further research is needed to identify factors that can refine the creatinine and BW-based predictive equation specifically for nonpregnant, nonlactating Holstein dairy cows.

## Highlights

• A fixed 29 mg/kg of BW creatinine rate estimated urine output within ±10% for 76.4% of the observations in nonpregnant, nonlactating Holstein cows.
• There was a bias estimate across BW quartiles.
• Model accuracy may improve by including factors such as body condition score and metabolic state.

## Graphical Abstract

**Figure:**
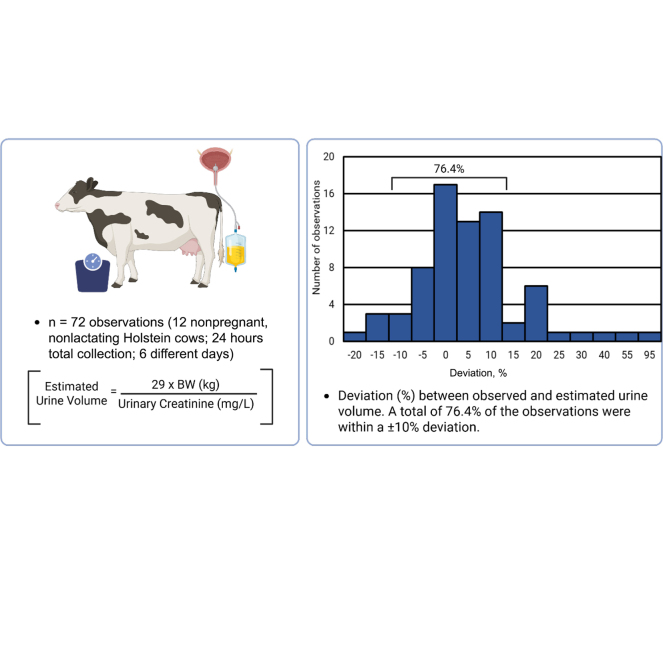
**Summary:** Urine analysis helps evaluate how cows use nutrients and excrete excess minerals and nitrogenous metabolites. Accurately estimating nutrient excretion requires both the concentration of urinary components and the total volume excreted. However, direct measurement of urine volume is labor-intensive and often impractical on commercial farms. This study evaluated a creatinine-based method to estimate urine output in nonpregnant, nonlactating Holstein cows (12 cows, 6 time points per cow, 72 observations). The estimated urine volume (EUV) was calculated using the following equation: EUV = [29 × BW (kg)]/[urinary creatinine concentration (mg/L)], where BW is body weight. There was a bias estimate across BW quartiles. Overall, 76.4% of the observations fell within a ±10% deviation range between estimated and observed urinary volume.

Urinary analysis can provide critical insights for optimizing nutrient utilization, refining feeding strategies, and promoting environmental sustainability (Husband and Vecqueray, 2007; Contreras-Jodar et al., 2019). Accurate assessment of kidney function requires determining both the concentration of excreted compounds, such as minerals (e.g., K, Na, S, Mg) or protein metabolism derivatives, as well as measuring total daily urine output (Nennich et al., 2006; Tebbe et al., 2018). However, conventional methods for determining urine volume in dairy cows, such as total collection via urinary catheterization, are invasive, labor-intensive, and impractical for large-scale or commercial applications due to logistical and animal welfare concerns (Tebbe and Weiss, 2018).

An alternative approach to overcome these limitations involves estimating daily urine volume based on the rate of creatinine urinary excretion relative to BW. Creatinine, a byproduct of muscle metabolism, is produced at a relatively constant rate, making it a useful marker for estimating urine volume (van Niekerk et al., 1963; Wyss and Kaddurah-Daouk, 2000). This estimation method has shown promising results for estimating urine output in lactating dairy cows (Tebbe and Weiss, 2018). A fixed creatinine excretion rate of 29 mg/kg BW has been validated for estimating daily urine output in lactating Holstein cows (Valadares et al., 1999; Tebbe and Weiss, 2018).

Despite its use in lactating cows, the applicability of this method in nonpregnant, nonlactating cows remains unclear. Therefore, this study aimed to evaluate whether the creatinine-based equation, which uses a fixed excretion rate, introduces bias when estimating daily urine volume in nonpregnant, nonlactating Holstein cows.

All animal-use procedures were approved by the Dairy Experts' Animal Welfare Committee (protocol number: DE20003). Data were obtained from a prior study that evaluated different sources of Mg and their bioavailability (Silva-del-Río et al., 2024). Briefly, the study included 12 nonpregnant, nonlactating Holstein cows assigned to one of two 6 × 6 Latin squares. Cow assignment to square was based on BW (square 1 = low BW and square 2 = high BW) and lactation number. Each experimental period lasted 7 d. Before the study, cows were moved to the research facilities and fed a basal diet for a 14-d adaptation period. On a DM basis, the basal diet consisted of corn silage (30.6%), oat hay (30.4%), and wheat straw (15.8%). The diet was designed to meet the animals' protein and energy needs of the animals using the professional version of the formulation software Nutritional Dynamic System (RUM&N Company, Reggio Emilia, Italy) with 12% CP. To avoid fluctuations in BW during the study, cows were offered 90% of the ad libitum DMI with unrestricted access to water.

In each experimental period, cows were weighed on d 2 and fitted with a 3-way urine latex catheter (24 French, 75 cc balloon; BD Foley, Franklin Lakes, NJ) for total urine collection on d 3 (24 h prior treatment) and d 4 (24 h after treatment). The catheter tubing was directed into an 18.9-L container containing 200 mL of glacial acetic acid (GL-7187; Stellar Chemical Corp., Rahway, NJ) to prevent mineral precipitation and biomolecule degradation. The observed urinary volume (**OUV**) was calculated from 24 h of total urine collection, with collection taking place every 12 h. After each collection interval, the container was emptied into a single 30-L portable milking bucket with engraved metric graduations used for all urine collections and stirring. The bucket was calibrated before the study by confirming the weights of 5, 10, and 15 L of water using a portable scale (QWORK; 220 ± 0.05 kg). All volumetric measurements during the study were performed by the same technician using the same calibrated bucket. An individual 3-mL urine sample was collected in a 5-mL cryovial tube with a screw cap and frozen at −20°C within 30 min of collection. To avoid the possible confounding effects of treatments on urinary output, we only included the baseline urinary output from weekly collections on d 3 and BW measurements on d 2 for this study. Furthermore, we tested the carryover effect of Mg treatments on urine volume, and it was found not significant (*P* > 0.35).

Urine samples of each 12-h collection period were analyzed for creatinine concentration at Michigan State University Veterinary Diagnostic Laboratory (Lansing, MI) using a colorimetric commercial kit (#OSR6178, Beckman Coulter, Crea, CA) with an intra-assay CV of 6.7%. Daily creatinine excretion was calculated by multiplying the urine creatinine concentration by the total urine volume collected during each of the 2 12 h periods (d 3) and adding the results; 72 daily observations were obtained. Estimated urinary volume (**EUV**) was calculated using the formula originally proposed by Valadares et al. (1999) and further validated by Tebbe and Weiss (2018): estimated urine volume = 29 × BW (kg)/creatinine (mg/L).

To evaluate the agreement between EUV and OUV, we developed 2 mixed-effects models using R statistical software (v4.3.2; https://www.r-project.org), both with mean-centered OUV as a fixed effect and cow as a random effect. The first model, with EUV as an outcome, assessed slope bias, defined as the deviation of the slope from a perfect linear relationship (slope ≠ 1) between EUV and OUV. The second model, which used the difference between EUV and OUV as the outcome, evaluated mean bias (average difference between EUV and OUV) by assessing whether the intercept deviated from zero (i.e., intercept ≠ 0). Additionally, due to the relationship used between BW and creatinine excretion, we evaluated the effect of BW on mean bias by including a variable categorized by quartiles (**BWQ_25_** [25th percentile; <655 kg], **BWIQR** [interquartile range 25th to 75th, 655 to 734 kg], and **BWQ_75_** [75th percentile; >734 kg]). Body weight was considered a confounding factor if, as a variable that might affect the estimated relationship between EUV and OUV, its inclusion in the model changed the estimated intercept by 10% or more. Degrees of freedom were calculated using the Kenward–Roger method (Kenward and Roger, 1997). The significance level for detecting differences among methods was set at *P* < 0.05, and the trend toward significance was 0.05 ≤ *P* ≤ 0.10.

Deviation between EUV and OUV. The deviation between EUV and OUV was calculated as deviation = (EUV − OUV)/OUV × 100. A Bland–Altman plot was used to calculate the CI for 95% of the mean deviation, with a focus on its relationship to BW. This analysis was performed using Python (version 3.13, Python Software Foundation). To complement the Bland–Altman analysis, the deviation between EUV and OUV was plotted as a histogram with intervals of 5% using Excel spreadsheets (Excel [Version 2411], Microsoft Corporation).

Daily OUV, urine creatinine concentration, urinary creatinine excretion, and daily creatinine excretion per kilogram of BW are presented in Table 1. The OUV ranged from 10.5 to 34.0 L/d and had a CV of 22.54%; 2 observations exceeded 3 SD from the average OUV from the sample mean but did not exceed 1.5 SD from the cow's mean and were retained for analysis. Urine creatinine concentration ranged from 573 to 1,551 mg/L, and urine creatinine excretion ranged from 9,879 to 27,481 mg/d. Daily creatinine ranged from 15.0 to 35.6 mg/kg BW with an average of 27.6 mg/kg BW. The EUV ranged from 12.62 to 36.39 L/d.

**Table 1:** Descriptive statistics of BW, urine output, and creatinine of the cows used in the study (n = 72 observations, 12 cows)

Variable	Mean	SD	Median	Minimum	Maximum
BW, kg	698	61.6	696	590	831
EUV,^1^ L/d	18.8	4.0	18.1	12.6	36.4
OUV,^2^ L/d	17.8	4.02	17.25	10.5	34.0
Creatinine, mg/d	19,301	2,807	19,177	9,879	27,481
Creatinine, mg/L	1,118	222	1,105	573	1,551
Daily creatinine, mg/kg BW	27.6	3.3	27.9	15.0	35.6

1 Estimated urine volume [urine volume = 29 × BW (kg)/creatinine (mg/L)].

2 Observed urine volume.

The slope of the first model between EUV and OUV was significantly different from 1 (slope = 0.86; *P* = 0.03; Figure 1), indicating proportional bias. Additionally, the intercept differed from the average OUV (18.79; *P* < 0.001), suggesting a baseline overestimation. The second model confirmed the presence of a mean bias between EUV and OUV (bias = 0.97 ± 0.32 L; *P* = 0.01). This bias varied by BW quartile: Cows in BWQ_25_ showed a negative bias (−10%), BWIQR showed no difference (0%), and BWQ_75_ showed a positive bias (+8%), indicating that bias varies across BW ranges. The deviation between EUV and OUV is presented as a modified Bland–Altman plot and a histogram (Figure 2). The mean deviation was 6.6%, with a 95% CI ranging from −24.0% to 37.3%. Overall, 76.4% of the observations fell within ±10%, and 83.6% within ±15% of the OUV.

**Figure 1:**
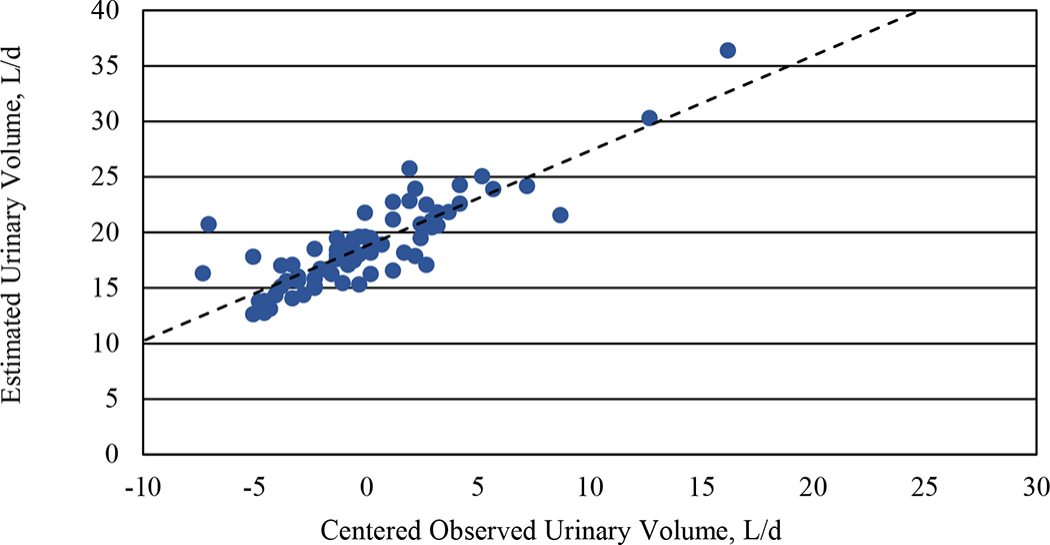
The relationship between mean-centered observed urinary volume (OUV) and estimated urinary volume (EUV) using creatinine in urine as a marker: Y = 0.86 (± 0.07)X + 18.79 (±0.31); R^2^ = 0.725; *P* < 0.01. The Y-intercept of the model was different from the OUV (*P* < 0.001), and the slope was different from 1 (*P* = 0.028).

**Figure 2:**
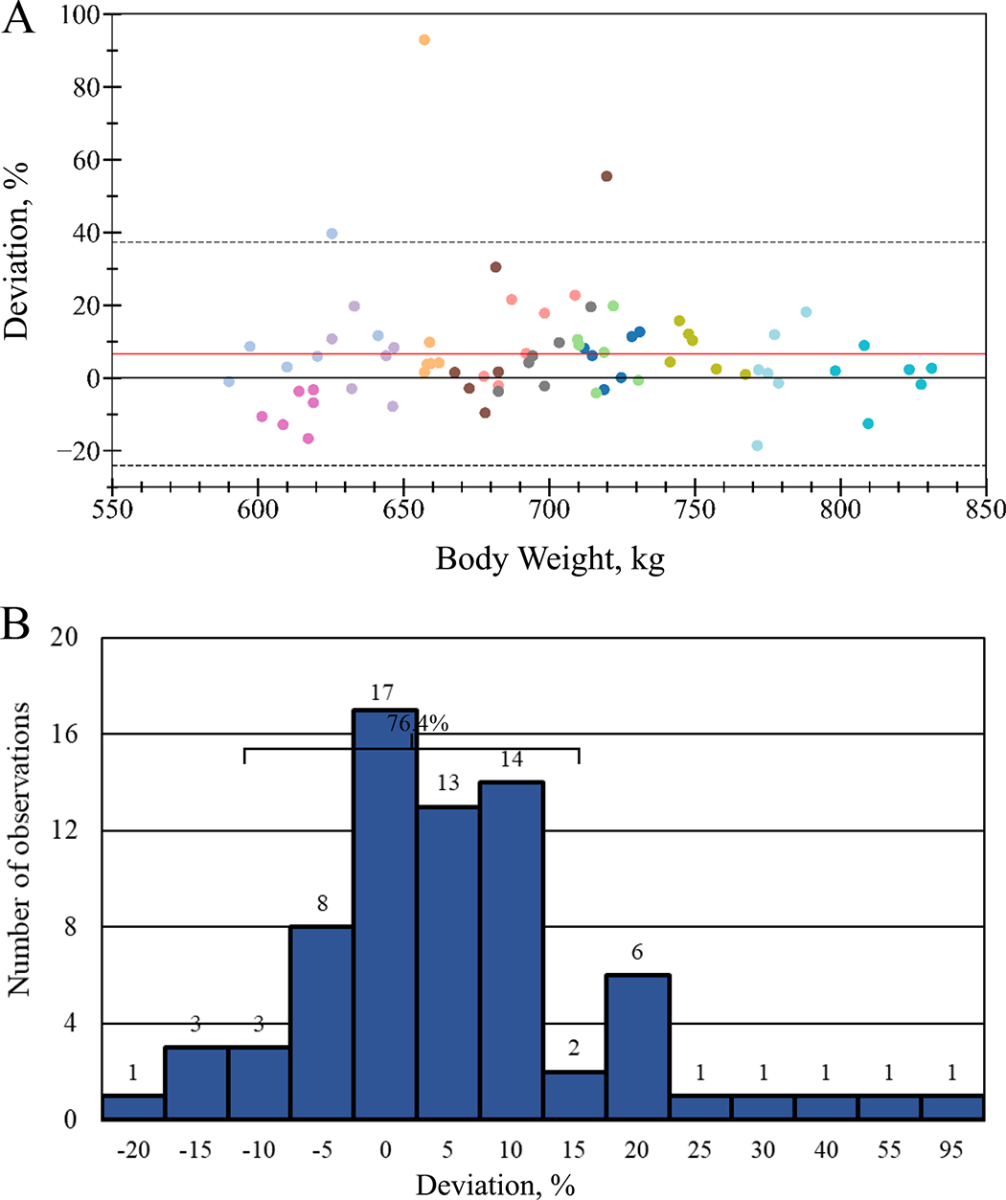
Deviation (%) between methods to determine urine volume excretion [deviation = (estimated urine volume − observed urine volume)/observed urine volume × 100] is represented here. (A) A modified Bland–Altman plot. The red and black lines represent the mean bias (+6.6%) and no bias, respectively. Dashed lines represent the ±1.96 SD limits of agreement. Each color represents a cow. (B) A bar distribution plot. Each bar represents a 5% increment. A total of 76.4% of the observations were within a ±10% deviation.

This study evaluated the accuracy of a creatinine-based equation to estimate urinary volume in nonpregnant, nonlactating dairy cows. The equation was previously proposed in lactating Holstein cows (Valadares et al., 1999). However, given the physiological differences between lactating and nonlactating animals, it was unclear whether the equation would perform similarly in nonlactating cows. Our findings suggest that while the equation provides a practical tool for estimating group-level urine output, it exhibits both proportional and mean bias, with BW as a contributing factor to mean bias.

The relationship between BW and creatinine excretion has long been used to assess urinary output and kidney function in veterinary medicine (Bovée and Joyce, 1979; Klante et al., 1997). This approach assumes that creatinine production is steady and proportional to muscle mass (Wyss and Kaddurah-Daouk, 2000; Dong et al., 2023). Based on this principle, Valadares et al. (1999) averaged urinary creatinine excretion across periods in 24 multiparous lactating Holsteins and proposed the 29 mg of creatinine/kg of BW coefficient. Tebbe and Weiss (2018) confirmed the same value in 17 multiparous Holsteins (139 ± 35 DIM; average BW of 713 kg). Other researchers have used this coefficient to estimate the daily urine volume in lactating dairy cows (Lee et al., 2012). Nonetheless, daily creatinine excretion is influenced by physiological factors such as metabolic state, muscle mass, and lactation status (Albin and Clanton, 1966; Sun et al., 2017; Whittet et al., 2019; Hanno et al., 2025).

In our study, the average daily creatinine excretion was 27.6 mg/kg BW, which falls within the range of 24.1 to 29.0 mg/kg BW as reported in previous studies with lactating Holstein cows (Valadares et al., 1999; Chizzotti et al., 2008; Tebbe and Weiss, 2018). The aforementioned studies included cows at varying stages of lactation (98 to 280 DIM), with BW ranging from 497 to 736 kg, and diets formulated to meet requirements for lactating Holstein cows. In contrast, Asai et al. (2005) reported an average creatinine excretion of 22.8 mg/kg BW in dry, pregnant Holstein cows, based on a small sample of 6 cows with urine collection performed at 3 time points during the dry period. Differences in creatinine excretion across studies may be partially explained by factors such as lactation status, pregnancy, diet composition, and BW.

Substantial cow-to-cow variation in creatinine excretion has been previously documented (Lee et al., 2019; Letelier et al., 2024). Contributing factors may include inherent individual differences (Albin and Clanton, 1966; Letelier et al., 2024), physiological stage (Chen and Ørskov, 2004), muscle catabolism associated with negative energy balance (Pires et al., 2013; Hanno et al., 2025), and differences in BCS (Roche et al., 2013; Jardstedt et al., 2017). Consequently, whereas creatinine-based equations may yield reasonable estimates at the group level, their precision at the individual cow level might be limited.

Because BW is part of the predictive equation for EUV, short-term fluctuations can introduce additional error. In this study, EUV was calculated using a single BW measurement per period. Although BW was recorded at a standardized time, normal variation in rumen fill or hydration can still produce within-cow variability. To assess whether this contributed to the observed bias between EUV and OUV, we recalculated EUV using each cow's mean BW across all 6 periods. The results were almost identical (mean EUV–OUV difference: −0.972 L using weekly BW vs. −0.960 L using average BW), indicating that short-term BW variation likely contributed minimally to random error. Therefore, the proportional and mean biases observed are unlikely to be driven by BW instability and more likely reflect biological variation in creatinine production or urine output. Therefore, the detected biases are likely due to biological variation in creatinine production or urine output rather than BW instability. Future predictive models may benefit from including additional cow-level covariates, such as BCS or metabolic status, to improve individual-level accuracy.

In addition to variability in creatinine excretion, urine output itself varied widely, ranging from 10.5 to 34.0 L/d. This is consistent with earlier studies reporting ranges of 17.9 to 44.3 kg/d (Lee et al., 2019), 18.4 to 50.6 kg/d (Letelier et al., 2024), and 13.1 to 33.1 kg/d (Spek et al., 2013). Urine output is influenced by dietary factors such as protein intake (Murphy, 1992) and physiological status, including hydration and renal function (Khelil-Arfa et al., 2012). However, in our study, all cows were fed the same diet and restricted to 90% of ad libitum intake with unrestricted access to water. Despite this, we observed 2 high output values (30.5 and 34.0 L/d) from the same cow, suggesting that variation in urinary excretion may exist even under controlled conditions.

Urinary creatinine concentrations in this study were higher than values typically reported for lactating Holstein cows, ranging from 554 to 1.062 mg/L (Danese et al., 2024; Padberg et al., 2025). This observation is consistent with the lower urine output of our nonpregnant, nonlactating cows (mean OUV = 17.8 kg/d) enrolled in our study, compared with the substantially higher urinary outputs documented for lactating cows (approximately 27–40 kg/d).

In our study, OUV was based on a single 24-h urine collection per period, which may introduce random error if a large urination event occurred near the start or end of the collection window or if an event was missed. Although multi-day collections are recommended to reduce this variability (Letelier et al., 2024), our design required weekly catheterization. Thus, extending catheterization duration posed animal welfare concerns. To evaluate whether single-day collections introduced meaningful error, we examined the stability of daily creatinine excretion, which should remain relatively constant within cow. Creatinine output was consistent for most animals (CV ≤10.1% for 9 of 12 cows). Among the cows with the highest CV values, 2 had single observations with unusually low creatinine output that might reflect missed urination events. However, none exceeded ±3 SD from the cow-level mean, so they were retained in the analysis.

Overall, this study provides valuable data for creatinine-based urine-based estimation in a previously untested population. Methodological strengths include a controlled diet and intake regimen that minimizes confounding effects in urinary excretion. Nevertheless, further research should aim for model refinement by incorporating variables that affect creatinine excretion in nonpregnant, nonlactating cows to reduce bias and improve utility.

Although the average creatinine excretion in our cows (27.6 mg/kg BW) was slightly below the fixed 29 mg/kg BW coefficient, it remained within the range reported for lactating Holsteins. Using this coefficient, 76.4% of EUV estimates were within ±10% of OUV, suggesting the equation can provide practical group-level estimates in nonpregnant, nonlactating cows. However, the proportional and mean biases observed, together with individual variability in creatinine excretion, limit its precision for individual cows. The equation may be useful for herd-level nutrient screening or treatment comparisons where bias is consistent across groups, but caution is needed when using it for individual-cow assessments or research requiring precise nutrient balance estimates.
